# In Favor of Controlling Proven, but Not Probable, Causes of Cancer: Landrigan et al. Respond

**DOI:** 10.1289/ehp.1104201R

**Published:** 2011-11-01

**Authors:** Philip J. Landrigan, Carolina Espina, Maria Neira

**Affiliations:** Mount Sinai School of Medicine, New York, New York, phil.landrigan@mssm.edu; World Health Organization, Geneva, Switzerland

We thank Erren et al. for their positive comments about our editorial on environmental and occupational causes of cancer (Landrigan et al. 2011). In particular, we acknowledge their support of our central thesis, expressed in the Declaration of Asturias [World Health Organization (WHO) 2011], that control of the toxic chemical causes of cancer must be a core component of global cancer control programs, equal in importance with efforts to understand and control “lifestyle” carcinogens such as diet, alcohol, and tobacco.

Erren et al. assert that programs aimed at control of chemical carcinogens must focus solely on chemicals that have been designated by the International Agency for Research on Cancer (IARC) as proven (class 1) human carcinogens (IARC 2011). Clearly class 1 carcinogens such as asbestos, benzene, benzidine, and dioxin merit very high priority in cancer control. There is no excuse, for example, for the continuing export of any form of asbestos to low- and middle-income countries.

We are of the view, however, that programs for control of chemical carcinogens must also encompass certain judiciously chosen class 2A or “probable” human carcinogens, such as diesel exhaust, indoor emissions produced by combustion of biomass fuels, and dimethylnitrosamine, for which there is already strong evidence of carcinogenicity in animal, cellular, or molecular models and limited human data (IARC 2011). In years past it would have been a serious lost opportunity not to have taken actions to control such carcinogens as formaldehyde or 1,3-butadiene during the years in which those compounds were classed by IARC as “probable” human carcinogens before they were upgraded to class 1.

For the future, as incidence rates of cancer and cardiovascular disease (CVD) continue to increase worldwide with accelerating global spread of the “Western lifestyle” and concomitant global diffusion of toxic synthetic chemicals, it will be imperative that disease control programs in countries around the world address both lifestyle as well as chemical causes of chronic illness. Strong synergies have been documented between lifestyle and toxic chemical exposures, for example, between cigarette smoking and asbestos in causation of lung cancer (Selikoff et al. 1968) and between diet and urban air pollution in causation of CVD (Brook and Rajagopalan 2010). Approaches to disease causation therefore need to address both lifestyle and toxic chemicals as causes of illness if they are to be fully effective in improving health and saving lives.
